# Epidemiology of Rotavirus-Norovirus Co-Infection and Determination of Norovirus Genogrouping among Children with Acute Gastroenteritis in Tehran, Iran

**DOI:** 10.22045/ibj.2016.05

**Published:** 2016-11

**Authors:** Seyed Dawood Mousavi Nasab, Farzaneh Sabahi, Manoochehr Makvandi, Siamak Mirab Samiee, Seyed Alireza Nadji, Mehrdad Ravanshad

**Affiliations:** 1 Department of Virology, Faculty of Medical Sciences, Tarbiat Modares University, Tehran, Iran; 2 Department of Medical Virology, School of Medicine, Ahvaz Jundishapur University of Medical Science, Ahvaz, Iran; 3 Reference Health Laboratories Research Center, Ministry of Health and Medical Education, Tehran, Iran; 4 Virology Research Center, National Research Institute for Tuberculosis and Lung Disease (NRITLD), Shahid Beheshti University, Tehran, Iran

**Keywords:** Gastroenteritis, Rotavirus, Norovirus, Coinfection, Epidemiology

## Abstract

**Background:**

Enteric viruses, particularly human rotavirus and norovirus, have been shown to replace bacteria and parasites, as the most common pathogens responsible for acute diarrhea. However, there are still few epidemiological data on the simultaneous occurrence of these viruses in Iran. In this regard, the aim of this study was to assess the useful epidemiological data on the gastroenteritis associated with rotavirus-norovirus mixed infection and to examine the prevalence of norovirus genogrouping among children aged less than five years old in Iran.

**Methods:**

A total of 170 stool samples were collected from children under five years of age with the clinical signs and symptoms of acute gastroenteritis, from May 2013 to May 2014. For the detection of both rotavirus and norovirus, total RNA was extracted from all samples, followed by reverse transcription polymerase chain reaction (RT-PCR). For both detected rotaviruses and noroviruses, genogrouping was performed.

**Results:**

Of 170 samples, 49 (28.8%) and 15 (8.8%) samples were found to be positive for rotavirus and norovirus infections by RT-PCR. Interestingly, 6 (3.5%) patients were positive for both infections. Among the 15 norovirus-positive patients, 13 (86.6%) and 2 (13.3%) belonged to genogroups GII and GI.

**Conclusion:**

The norovirus genogroup GII and rotavirus lead to the serious infections in children with acute gastroenteritis. However, more well-designed studies are needed to further elucidate the role of other enteric viruses in acute gastroenteritis

## INTRODUCTION

Acute gastroenteritis is a global public health problem caused by bacteria, viruses or various parasites. The disease is occurred among children under five years of age, with an estimated 1.5 billion episodes of diarrhea and 65,000 deaths annually in 22 countries of the Eastern Mediterranean Region[1,2].

The findings have shown that enteric viruses are the most significant pathogens in acute gastroenteritis, as compared with other pathogens[3]. The most important viruses involved in outbreaks of enteric gastroenteritis include rotavirus, human norovirus, adenoviruses 40 and 41 and astrovirus[4,5].

Rotaviruses have double-stranded RNA (dsRNA) genomes and belong to the family *Reoviridae*. Based on RNA divergent sequence in VP6 region, rotaviruses are classified into eight groups (A–H)[6]. Rotavirus Group A is predominantly associated with gastroenteritis in infants and young children[7]. The segmented dsRNA genome of rotavirus is enclosed in a triple-layered capsid structure. The outer capsid is composed of proteins, VP7 (G-type [glycoprotein]) and VP4 (P-type [protease-sensitive protein]), which display neutralization properties and are used for viral classification[8]. At present, at least 27 G genotypes (15 of which corresponding to G serotypes) and 35 P genotypes (14 of which corresponding to P serotypes) have been documented[9].

Rotavirus infections have been estimated to account for 111 million cases of acute gastroenteritis requiring home care services and 25 million cases requiring referral to a physician each year. Rotavirus gastroenteritis is annually responsible for nearly 2 million hospitalizations and 527,000 deaths in children up to five years of age worldwide[10,11]. In developed countries, rotavirus vaccine coverage is high enough to provide herd immunity and to induce protective responses against rotavirus infection in most immunized children[12]. However, in developing countries, rotavirus vaccination has not been implemented. For example, in Iran, vaccination against rotavirus has not been included in the national immunization program. Several epidemiological surveys carried out in Iran have provided evidence that rotavirus infection rates range from 11.36% in Shiraz to 79% in Tehran[13-15].

Noroviruses are the most common cause of acute non-bacterial gastroenteritis outbreaks in children worldwide[16]. Norovirus gastroenteritis has recently been reported to be the second cause of children’s hospital admissions, after rotavirus gastroenteritis[17-19]. It has also been reported that acute gastroenteritis due to noroviruses results in 200,000 child deaths every year in developing countries[17-19]. Norovirus is a member of the family *Caliciviridae* containing a 37-nm diameter genome. It is also a single-strand, positive-sense virus with three open reading frames (ORFs) consisting of non-structural polyproteins, such as RNA polymerase (ORF1), major capsid protein (ORF2) and minor structural protein (ORF3)[20,21]. Based on the partial sequence of the RNA genome and genomic heterogeneity, noroviruses are divided into genogroups GI through GVI. Genogroups G1 and G2 are further divided into 9 and 22 genotypes, respectively[22,23]. Importantly, only genogroups GI, GII and GIV are able to infect humans, while GI and GII are considered to be the most prevalent norovirus infections in human beings[24].

The norovirus genomic RNA has the intrinsic capability to recombine or mutate, thus facilitating the emergence of new variants[25]. Therefore, it is difficult to prevent and control norovirus infections[26,27]. Different seasonal patterns of norovirus infections have been observed all over the world[20,23].

The involvement of rotavirus and norovirus in children with acute gastroenteritis and the epidemiological feature of either rotavirus or norovirus infections has been documented in Iran[28-30]. The present study was conducted to evaluate the association between rotavirus and norovirus infections and to determine norovirus genogroup in children with acute gastroenteritis, which have not been reported yet in Iran.

## MATERIALS AND METHODS

### Definition of diarrhea

A day with diarrhea was determined by the occurrence of three or more liquid or semiliquid stools during a 24-h period.

### Fecal sample collection

A total of 170 diarrheic stool samples (130 outpatients and 40 inpatients) were collected from children (under five years of age) with acute gastroenteritis. The cases were referred to Children’s Medical Centers in Tehran, Iran from May 2013 to May 2014. The patients’ symptoms were accompanied with or without vomiting, fever, nausea, abdominal pain and cramp. Meanwhile, the absence of leukocytes, red blood cells and pus in the stool was confirmed by microscopic examination. The stool samples were then transported on ice to the Virology Department at Tarbiat Modares University (Tehran, Iran) and stored at -20°C until process.

### Ethics statement

This study was approved by the Ethics Committee of Tarbiat Modares University, and an informed consent was obtained from the mothers of the patients.

### Viral RNA extraction

To extract RNA, approximately 10% (w/v) suspension of each stool sample was prepared. Briefly, 1 g (pea-sized) or 100 μl stool from each patient was dissolved in 1000 µl phosphate buffered saline and centrifuged at 400 ×g for 20 minutes. Next, 200 μl supernatant from each sample was collected for RNA extraction using the RTP^®^ DNA/RNA Virus Mini Kit (Stratec Biomedical AG, Germany) according to the manufacturer’s instructions. RNA extracts were stored at -70°C until use.

### Reverse transcription

Reverse transcription polymerase chain reaction (RT-PCR) was conducted with the RevertAid RT Reverse Transcription Kit (Thermo Fisher Scientific,

USA) according to the manufacturer’s instructions. Briefly, RT reactions were carried out in a final volume of 20 μl containing 4 μl 5× RT buffer, 1 μl 10 mM dNTPs, 1 μl 0.2 U/μl random hexamer, 1 μl 40 U/μl RNase inhibitor, 1 μl 200 U/μl RT enzyme, 6 μl DEPC water (RNase free water) and 6 μl extracted RNA. The reactions were incubated at 42°C for 1 h. To facilitate the reverse transcription of rotavirus dsRNA, the mixture of the primer and template was first denatured by heating at 95°C for 5 min and then snap-chilled on ice for 1 min. The cDNA samples were stored at -20°C until use in PCR reaction.

### Rotavirus detection

As shown in Table 1, one oligonucleotide primer pair was used to detect rotavirus. The PCR reaction was performed in a final volume of 25 μL containing 4 μL 10× PCR buffer (CinnaGen, Iran), 0.5 μl each 10 pmol/μl primer, 1 μl 10 mM dNTPs (Fermentas, USA), 0.3 μl 500 U/μl Taq DNA polymerase (CinnaGen), 0.5 μl 50 mM MgCl_2_ (CinnaGen), 11.2 μL H_2_O and 7.0 µl cDNA template. PCR cycles were carried out in a thermal cycler (Applied Biosystems GeneAmp® verity thermocycler) as follows: an initial denaturation at 95°C for 5 min, 40 cycles of denaturation at 94°C for 3 min, annealing at 55°C for 55 s, elongation at 72°C for 1 min and a final extension step at 72°C for 7 min. The amplification of 569-bp PCR product was considered as positive.

**Table 1:** The sequences of primers used for detection of rotavirus Group A and norovirus genogrouping

Virus	Polarity	Sequence (5’-3’)	Product size (bp)	Gene
Rotavirus	Forward	AAA GGA TGG CCA ACA GGA TCAT	569	*VP7*
	Reversed	GTA TAR AAH ACT TGC CAC CAT		
Norovirus G1	Forward	CTG CCC GAATTY GTA AAT GA	330	*ORF2*
	Reversed	CCAACC CARCCATTR TAC A		
				
Norovirus GII	Forward	CAR GAR BCNATGTTYAGRTGGATG AG	388	*ORF2*
	Reversed	CCR CCN GCA TRH CCR TTR TAC AT		
Norovirus GIV	Forward	GCACTCGGCATCATGACAAAATTCA	995	*ORF1/ORF2*
	Reversed	GTTTGGGTCCCAATTCCAA		

### Detection of genogroups GI, GII and GIV

To detect genogroups G1, G2 and G1V among patients with acute gastroenteritis, three sets of primers were applied for ORF2 and ORF1/ORF2 regions. The PCR reaction was carried out as above. The sequences of the primers, amplicon and target genes are illustrated in Table 1[31-33]. The resulting PCR products were analyzed on 2% agarose gels, stained by the GelRed dye (GelRedΤΜ Nucleic Acid Gel Stain) and visualized under UV light. The products were then extracted using a QIA quick PCR Purification Kit (Qiagen, Hilden, Germany). To confirm positive PCR results, the PCR products were sequenced (Macrogen, South Korea). Afterwards, the positive sequences were edited and aligned with those deposited in the GenBank database using the BioEdit software (version 7.0.5.2.) and Clustal X (version 2.0), respectively.

### Statistical analysis

Analysis of the data was performed by the SPSS statistical software version 21.0. Chi-square (χ^2^) test was used to compare the groups, and the test was used for each virus separately. *P* value less than 0.05 was considered to be statistically significant.

## RESULTS

Out of 170 samples, 54.1% were male and 45.9% were female. All the samples were found to be negative for the bacteria and parasites. Demographic parameters, including age, gender and the season of sample collection were recorded for each patient (Table 2).

**Table 2:** The characteristics of positive patients studied

Parameter	Rotavirus (n=49)		Norovirus (n=15)		*P* value	Rotavirus-norovirus (n=6)
	
	n	%	n	%		
Gender					
Female	19/170	11.17	5/170	2.9	Rotavirus (0.237)	1/6 (16.6%)
Male	30/170	17.64	10/170	5.9	Norovirus (0.307)	5/6 (83.3%)
Age (montd)					
>12	15	30.6	4	26.7	Rotavirus (0.005)	0
13- 24	23	46.9	6	40		4
25- 36	8	16.3	3	20		2
37- 60	3	6.1	2	13.3		0
Season					
Spring	5	10.2	3	20	Rotavirus (0.0001)	2
Summer	2	4	0	0		0
Autumn	10	20.4	7	46.7		1
Winter	32	65.3	5	33.3		3

**Fig. 1:**
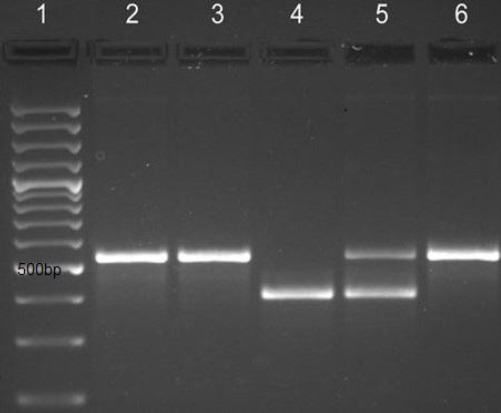
Detection of rotavirus and norovirus using PCR. Lane 1, 100 bp ladder; Lanes 2, 3 and 6, the amplified fragment related to rotavirus (570 bp); Lane 4, the amplified fragment of norovirus (330 bp), Lane 5, rotavirus-norovirus co-infection.

RT-PCR analyses indicated that among 170 samples, 49 (28.8%) and 15 (8.8%) were positive for rotavirus and norovirus infections, respectively. However, 6 (3.5%) samples were positive for both rotavirus and norovirus (Table 3). Figure 1 shows the positive PCR results on the agarose gel electrophoresis. The authenticity of the PCR product was confirmed by sequencing. A higher rate of rotavirus infection was detected in males than females, which was not significant (95%CI=1.503, 0.764-2.955). In rotavirus-infected group, the highest prevalence was observed in children between 13 and 24 months of age, including 46.9% of all positive cases (95%CI=2.683, 1.337-5.384). Rotavirus was also detected throughout the year. The prevalence of rotavirus gastroenteritis was 10.2% in spring, 4% in summer, 20.4% in autumn and 65.3% in winter. The noroviruses-infected group (n=15) had 3 patients in spring (20%), no patient in summer, 7 in autumn (46.7%) and 5 in winter (33.3%). A significant difference was observed between the incidence of rotavirus in cold season with that of the rest of the year, as demonstrated by the chi-square test (95%CI= 5.465, 2.671–11.182) (*P<*0.0001).

**Table 3:** The detection rate of rotavirus and norovirus or dual positive infections in inpatient and outpatient stool specimens

Virus	Total no. of cases (%)	Inpatient (%)	Outpatient (%)
Rotavirus	49 (28.8)	10/40 (25)	39/130 (30)
Norovirus	15 (8.8)	4/40 (10)	11/130 (8.5)
Norovirus-rotavirus	6 (3.5)	4/40 (10)	2/130 (1.5)

**Fig. 2:**
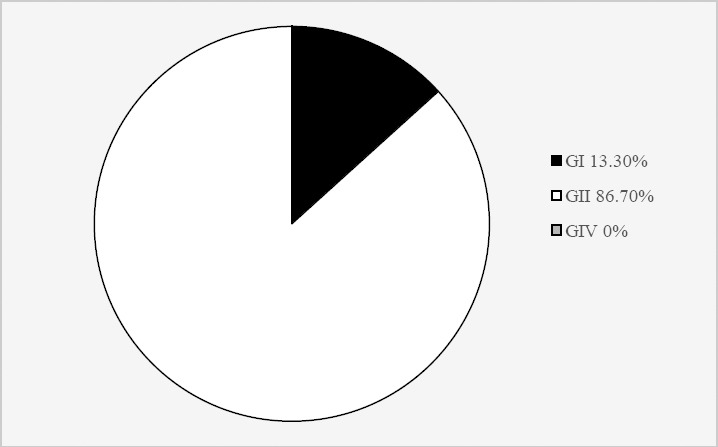
The prevalence of norovirus genogroups

The frequency of diarrhea for norovirus was significantly higher in males (95%CI=1.780, 0.582–5.451) (*P*=0.307), as compared to the females. Children less than 24 months of age accounted for 70.83% of the overall norovirus-positive cases with those between 13 and 24 months of age being the most affected (*P*=0.214). The majority of rotavirus-norovirus dual infections were detected in winter and observed in children with an overall median age of 18 months (Table 2). Among the 15 norovirus-positive children, 13 (86.7%) and 2 (13.3%) belonged to genogroups GII and GI; however, genogroup GIV was not detected in this study (Fig. 2).

Among the 130 outpatients, 39 (30%) and 11 (8.5%) cases were positive for rotavirus and norovirus, and rotavirus-norovirus co-infection was observed only in two cases. Among the 40 hospitalized patients, 10 (25%) and 4 (10%) cases were found to be positive for rotavirus and norovirus and four patients had rotavirus-norovirus (genogroup GII) mixed infections.

## DISCUSSION

Diarrhea remains the second leading cause of death due to infections among children under the age of five years worldwide[34]. Many reports have established the importance of rotaviruses and noroviruses as the causes of outbreaks of gastroenteritis[1,2,7,49]. In the present study, we have determined the prevalence of rotavirus and norovirus infections and co-infections among the children with acute gastroenteritis. To our knowledge, this is the first report to determine the human norovirus genogroups (G1, GII and GIV) among children with acute gastroenteritis in Iran.

In this study, rotaviruses were detected in 28.8% of the patients with acute gastroenteritis, which is consistent with the results reported by Kargar *et al*.[35] and Parashar *et al*.[36]. Our findings are also in line with the results of Shoja *et al*.[13] and Moradi-Lakeh *et al*.[14], who reported 11.36% and 79% of rotavirus in two different regions of Iran. The difference between the rate of rotavirus detection among the male (17.64%) and female populations (11.17%) was not significant (*P*<0.237).

The peak of rotavirus infections in Latin America was proclaimed to be in autumn and winter[37]. The findings of the present study also demonstrated that the peak of rotavirus infections occurs in the winter season (*P*<0.0001). Also, the highest rate of rotavirus infection was observed in children between 13 and 24 months of age (*P*<0.005), which was similar to another study[38].

Norovirus is considered as the second most common cause of viral gastroenteritis with a prevalence rate of 6%-19% globally. In this survey, of 170 patients, 15 (8.8%) cases had positive norovirus infection, which is in agreement with the results of other studies reported in Iran[39-41]. In the present study, noroviruses were detected in spring, autumn and winter but not summer. There was also a considerable increasing trend in the number of cases in the autumn season, which confirms the previous studies[42,43]. The highest rate of norovirus infection was observed in children under 24 months (40%), which is in accordance with the studies reported from Iran[40], UK[44] and China[45]. The prevalence of norovirus detection in males (10.8%) and females (6.4%) is the same as the results reported from Japan by Ozawa *et al*.[45]. Our findings revealed that norovirus accounts for 13% of genogroup GI and 87% genogroup GII in cases with acute gastroenteritis. This shows that norovirus genogroup GI is less common than norovirus GII and similar to a report by Bon *et al*.[18] from France, norovirus GII is the most dominant genogroup in our region. There are few reports available on detection of norovirus genogroup GIV[46,47]. In contrast to the norovirus genogroups GI and GII, the GIV genogroup was not detected in our study. The coinfection of rotaviruses and noroviruses has been reported by some investigators in different regions of the world. In a study conducted by Sai *et al*.[4] in China, the frequency of rotavirus and norovirus infections was 34.4% and 10.4%, respectively, and their coinfection was 1%. In Morocco, El Qazoui *et al*.[48] reported the frequency of rotavirus and norovirus and coinfection of rotavirus-norovirus among children less than 24 months of age to be 26.6%, 16.1% and 2.7%, respectively. Oldak *et al*.[49] studied rotavirus and norovirus among the patients with acute gastroenteritis in Netherland. They reported that 48.6% of the patients were positive for rotavirus and 16.5% for norovirus and 3.8% had rotavirus-norovirus coinfection.

In the present study, the coinfection of rotavirus and norovirus was found to be 3.5% and was mostly detected during the cold season, which is in consistent with the result of Tran *et al*.[50]. We also indicated that the norovirus genogroup GII and rotavirus lead to serious infections in children less than five years old with acute gastroenteritis. However, more well-designed studies are necessary to further elucidate the role of other enteric viruses in acute gastroenteritis.
